# Everyday attention and lecture retention: the effects of time, fidgeting, and mind wandering

**DOI:** 10.3389/fpsyg.2013.00619

**Published:** 2013-09-18

**Authors:** James Farley, Evan F. Risko, Alan Kingstone

**Affiliations:** ^1^Department of Psychology, University of AlbertaEdmonton, AB, Canada; ^2^Department of Psychology, University of WaterlooWaterloo, ON, Canada; ^3^Department of Psychology, University of British ColumbiaVancouver, BC, Canada

**Keywords:** fidgeting, time on task, lecture, memory, attention, mind wandering, ecological validity, cognitive ethology

## Abstract

We have all had our thoughts wander from the immediate task at hand. The emerging embodied cognition literature emphasizes the role that the body plays in human thought, and raises the possibility that changes in attentional focus may be associated with changes in body behavior. Recent research has found that when individuals view a lecture, mind wandering increases as a function of time. In the present study we asked whether this decline in attention during lecture viewing was associated with fidgeting. Participants were filmed while they watched a 40-min lecture video, and at regular 5-min intervals provided ratings of their attentiveness. Following the lecture, participant's memory for the material was assessed. Fidgeting behavior was coded from video recordings of each session. Results indicated that attention to, and retention of, lecture material declined as a function of time on task. Critically, and as predicted, fidgeting also increased with time on task. We also found that the relation between fidgeting and retention was significant even when the role of attention was factored into the equation, suggesting that fidgeting makes a unique contribution to retention of lecture material over and above that contributed by an individual's attention. We propose a novel non-attentional stress-based account of fidgeting and how this impacts retention for lecture material over and above changes in levels in mind wandering vis-a-vis changes in attention.

## Introduction

People spend a sizeable portion of their day-to-day lives mind wandering (i.e., engaged in some form of off-task thought; Killingsworth and Gilbert, 2010). This ubiquitous activity may be largely innocuous, or even beneficial (Mooneyham and Schooler, 2013; Franklin et al., 2013), but in many circumstances mind wandering can have deleterious consequences for the mind wanderer. One such circumstance that has received some attention recently is the academic lecture (Lindquist and McLean, 2011; Risko et al., 2012a, 2013a; Szpunar et al., 2013a). Lectures, live or video, are one of the primary means of knowledge transmission in post-secondary education (e.g., Bligh, 2000). Unfortunately, students spend much of their time in lectures mind wandering, and mind wandering has been demonstrated to impair memory for lecture material (Lindquist and McLean, 2011; Risko et al., 2012a, 2013a; Szpunar et al., 2013a,b). While this tendency to mind wander is not unique to lectures and appears to be a stable phenomenon across both laboratory experiments and a broad range of everyday activities (McVay et al., 2009), the costs may be particularly high in a lecture context. For example, lecture's cumulative nature might amplify the cost of momentary attentional lapses by disrupting a student's ability to form and maintain a situation model (see the cascade model of inattention; Smallwood, 2011). Thus, efforts to understand the factors which influence mind wandering, the cues that could indicate that a student is mind wandering, and, ultimately, the strategies that could be used to reduce mind wandering in lectures have the potential to both improve our understanding of attentional function in a naturalistic context and improve educational outcomes. In the present investigation we examine the effect that time on task has on attention to, and memory for, lecture material, in addition to exploring fidgeting as an overt cue of an individual's attentional state.

## Time on task

Listening to a lecture can be considered a kind of sustained attention task (Young et al., 2009; Risko et al., 2012a). There exists both a broad and deep literature on sustained attention (Mackworth, 1948; Parasuraman, 1985; Warm et al., 2008a,b; Smilek et al., 2010a; Seli et al., 2012). One of the classic phenomena explored in this literature is the vigilance decrement, which reflects the observation that performance in sustained attention tasks decreases as time on task increases (e.g., Mackworth, 1948). The tasks typically used to investigate the vigilance decrement require the detection of infrequent events over a long period of time (e.g., radar operations). While on the surface listening to lectures might be considered a different type of task, they share the need to sustain attention for an extended period of time and the need to monitor the lecture for relevant events (e.g., important points; Einstein et al., 1985; Risko et al., 2013b). As such, one might expect to find a pattern similar to a vigilance decrement in lectures. Evidence consistent with this notion has been reported recently (Young et al., 2009; Risko et al., 2012a, 2013a). For instance, in Risko et al. (2012a, 2013a), when a lecture was divided into “early” and “late” time periods, reports of mind wandering increased and memory for lecture material decreased from the early to late period. Thus, in this case, the ability to sustain attention in a lecture (as measured via mind wandering frequency) appears to be related to the time spent monitoring the lecture material. A number of potential mechanisms have been put forward to explain attentional variations over time (see Risko et al., 2012a). For example, according to a resource depletion account, sustaining attention draws on a limited resource and over time that resource dissipates until the ability to sustain attention is compromised. Alternatively, mind wandering might increase over time because task-relevant processes become automatized thus freeing up resources to devote to task unrelated thoughts.

Risko et al. (2012a, 2013a) also provided evidence that overall mind wandering frequency was correlated with memory for lecture material, a result consistent with much research demonstrating that mind wandering leads to encoding deficits (Manly et al., 1999; Smallwood et al., 2007a, 2008; Christoff et al., 2009). While some caution should accompany correlational evidence, the association between time on task, mind wandering frequency, and memory for lecture material provides a strong motivation to better understand variations in attention and memory within the lecture environment. In the present investigation, we extend this work by providing a finer grained evaluation of variations in attention and memory for lecture material over the course of a 40-min lecture. In addition, we explore a potential overt indicator of an individual's internal attentional state, by investigating how fidgeting relates to attention, time on task, and memory for lecture material.

## Shades of attentiveness

Research investigating mind wandering has begun to emphasize the importance of its graded nature. For example, one of the distinctions frequently made in the mind wandering literature relates to the level of awareness associated with mind wandering (Schooler et al., 2011). Mind wandering without awareness (often referred to as “zoning out” and typically indexed exclusively by “probe caught” reports of mind wandering) has been found to have somewhat different properties than mind wandering with awareness (often referred to as “tuning out” and typically indexed by “self caught” reports of mind wandering). Mind wandering without awareness, for example, has been found to be more strongly associated with response inhibition failures (Smallwood et al., 2007b). Similarly, “shallow” mind wandering can be contrasted with that of “deep” mind wandering. Smallwood (2011) used such a distinction in a model wherein varying levels of perceptual decoupling were associated with varying consequences for the processing of text. Similarly, Schad et al. (2012) demonstrated that different levels of mindless reading could be identified based on the nature of the errors noticed by the participants. Lastly, and importantly in the present context, there is reason to think that the graded nature of attentiveness will be an important consideration in the context of studying the relation between fidgeting and attention, as Carriere et al. (2013) found that fidgeting (as indexed by self-reports) was predicted by spontaneous mind wandering but not intentional mind wandering. To capture this graded nature of attentiveness, the present work utilized a continuous measure of attention (in the form of a 7-point Likert scale) rather than the more traditional dichotomized measure (i.e., mind wandering? yes/no).

## Fidgeting

People often use the overt behaviors of others in order to infer their internal mental state (e.g., Mehrabian, 1969). In a recent series of experiments Chisholm et al. (2013) demonstrated that leaning forward and back are viewed consistently as indicating focused and unfocused attentional states. Another overt cue commonly used to infer another person's attentional state is fidgeting (Galton, 1885; Mehrabian and Friedman, 1986; Gligoric et al., 2012; Carriere et al., 2013). In particular, there is a widely held notion that fidgeting is associated with faltering attention. For example, Gligoric et al. (2012) surveyed 230 students about their beliefs in what best exemplifies impoverished attention in a lecture setting. The respondents listed fidgeting (along with making noise) as one of the two most common indicators of waning attention in the classroom environment. In addition, Mehrabian and Friedman (1986) found a positive correlation between tendency to fidget (both self-reported and objectively coded) and propensity to daydream. In a more recent series of studies, using various self-report measures of fidgeting and attention, Carriere et al. (2013) also found a strong association between self-reported fidgeting behavior and decreased attention and mind wandering.

The embodied cognition literature emphasizes the central role the body plays in mediating cognition (see Wilson, 2002). In this context one potentially fruitful way to view fidgeting is as an “embodiment” of the act of sustaining attention. For example, Smilek et al. (2010b) found that increased rates of blinking were associated with increased rates of mind wandering (i.e., inattentiveness to the text being read). They interpreted this relationship as reflecting a mechanism by which the flow of sensory information is physically interrupted to facilitate a shift in the relative balance of internally and externally directed processing (i.e., more blinking reflects more internally directed attention). Thus, the physical act supplements the shift of attention. Likewise, fidgeting may represent an overt action that interacts with systems supporting the ability to sustain attention and thus act as a potential route to optimize attention. One possibility is that fidgeting might provide a form of a “mental break.” For example, Ariga and Lleras (2011) have demonstrated that task switches, in the context of a sustained attention task, can decrease the vigilance decrement effect (see also Mackworth, 1948). Fidgeting may also help individuals sustain attention by increasing physiological change and arousal. Levine et al. (2000) measured energy expenditure in a variety of non-exercise activities and found that sitting while fidgeting increased energy expenditure by 54% on average relative to sitting at rest. Indeed, the fidgets can be quite small yet still have a pronounced effect. For example, Andrade (2010) had participants doodle during a “boring” task and discovered that it improved performance which was attributed to an increase in overall arousal. Thus, on this account, fidgeting can be taken as providing a physical indicator of attentiveness in so far as its adoption reflects an attempt to combat waning attention. In a similar manner, fidgeting may provide a physical marker of the transition *into* a state of inattention (an increase in inattention; akin to an individual's averted gaze during a conversation) rather than an attempt to transition *from* a current state of inattention. Note that in either case fidgeting would reflect the presence of inattention.

This relation between fidgeting and attention makes the straightforward prediction that fidgeting should increase as a function of time spent viewing the lecture (i.e., given attention is known to decrease as a function of time in a lecture). In addition, variations in fidgeting should predict variations in attention (i.e., more fidgeting reflecting reduced attentiveness). The current study puts these predictions to the test.

## Present investigation

In the present investigation we explore the influence of time on task on attention, fidgeting, and memory within a lecture-viewing context. We also assess the relation between these factors. Participants viewed a 40-min lecture. After each 5-min increment individuals reported their attentional state using a continuous 7-point scale. Following the lecture participants completed a retention test composed of 3 questions from each 5-min increment in the lecture. Thus, the measurement of time on task's influence on memory for lecture material consisted of assessing fluctuations in memory for material presented during 5-min periods of the lecture. Lastly, each session was video recorded and from these recordings fidgeting was coded and binned into the corresponding 5-min intervals of the lecture. According to the discussion above, attention to the lecture should decrease as a function of time. In addition, if fidgeting reflects an attempt to stave off decreases in attentiveness, then it should increase as a function of time. Fidgeting should also be negatively correlated with attention, such that fidgeting should increase during blocks associated with reduced attentiveness.

## Methods

### Participants

Twenty-one undergraduate students (sixteen of which were female) from the University of British Columbia participated in exchange for course credit.

### Design

The primary independent variable was block. The lecture was divided into eight 5-min blocks resulting in a one-factor (block) design with eight levels. The dependent variables included attentional self-reports, scores for two types of fidgeting, and retention for lecture material across each block.

### Apparatus

Each participant viewed the lecture video individually within a media immersion room, seated approximately 6 feet in front of a big-screen television connected to an external stereo. A single ceiling mounted video camera recorded the full duration of each viewing session. Self-reports of attentional state were recorded on a nearby laptop, which sounded a tone to prompt for a response.

### Stimuli

The stimulus was a 40-min video recording of an academic lecture obtained from Open Yale Courses (http://oyc.yale.edu). This lecture was taken from the Introduction to Psychology course, titled “Evolution, Emotion and Reason: Love” (Salovery, 2009) and was delivered within a traditional classroom-based context. The content centered around the notion of love and interpersonal relations in general. It was selected to be of general interest to a broad range of participants.

### Self-reports of attentional state

The self-report of attentional state took the form of a 7-point Likert-type response to the statement “My attention is fully focused on the video.” Responses used the following labels: “Strongly Disagree,” “Somewhat Disagree,” “Slightly Disagree,” “Neither Agree nor Disagree,” “Slightly Agree,” “Somewhat Agree,” or “Strongly Agree.” Participants were advised that, instead of reporting their attentional state at the discrete point in time when prompted, responses should be based on their average attentional state throughout the preceding 5-min interval. Rather than being explicitly framed as indexing “mind wandering,” the scale was presented as reflecting a more generalized level of focus, and in doing so, we sought to capture the notion described in the introduction that there may be a gradation in mind wandering.

### Retention test

The test consisted of 24 questions, evenly distributed across each of the 8 blocks. The questions were recall in nature and answers typically consisted of single word concepts, key terms, or simple main findings. Accuracy for each question was simply scored as either being correct or incorrect. The questions were not cumulative in style in order to minimize the likelihood of earlier lapses in attention affecting future comprehension. The selection criteria for the retention questions largely related to the pace of the lecture in that three questions per 5-min block was approximated to be the maximum number of questions that could be consistently produced without referencing trivial points. The retention test was completed immediately following viewing of the lecture.

### Procedure

Participants were told that they would watch a lecture and answer questions about it once completed. In addition, participants were instructed to self-monitor fluctuations in their attentional state and report them using the scale provided when prompted. Following the lecture, participants received the retention test.

### Coding fidgeting

The fidgeting coding consisted of quantifying the total number of movements observed within each 5-min block. Coders worked their way sequentially through each participant one at a time. Each distinct movement was coded as belonging to one of three categories (a) head movements, (b) appendage movements, or (c) body movements. Furthermore, each movement was also coded as belonging to one of two levels of magnitude: macro or micro. The macro movements were operationalized as involving complete spatial displacement relative to a starting position (i.e., moving the arm to a completely new location, not overlapping that of the initial), whereas anything less was considered a micro movement. Values for each coding category were tabulated on a minute-by-minute basis by the coders, which were then converted into totals for each category corresponding to each individual block for every participant. The first author, JF, coded all data. In order to determine inter-rater reliability a second coder coded 25% of the videos and these results were correlated with JF's coding of the same videos. In order to determine intra-rater reliability JF re-coded the same 25% of the videos and these results were also correlated with JF's initial coding of those videos. Intra-rater reliability for micro and macro movements was good, *r* = 0.92, *p* < 0.05, and, *r* = 0.69, *p* < 0.05, respectively, as was inter-rater reliability for the same, *r* = 0.61, *p* < 0.05, and, *r* = 0.89 *p* < 0.05. We report analyzes for both micro and macro fidgeting. Collapsing across the micro/macro dimension to produce a singular fidgeting measure resulted in a qualitatively similar pattern of results (not reported). However, there were a few instances when the macro measure was a significant predictor while the micro was not. The opposite pattern did not occur. Additionally, in models for which both measures were predictive, the relationship was always stronger when considering macro fidgets. This suggests the micro/macro distinction is a useful way to parse fidgeting behavior and bolsters its predictive power. The overall correlation between macro and micro fidgets across all subjects was *r* = 0.45. Note that due to temporary camera misalignment, six participants were recorded with their legs positioned outside of the frame. Of course, given the within subjects design of the experiment, such a systematic underestimation should not change the pattern of results observed with respect to relative changes in frequency of fidgeting. Nevertheless the fidgeting data were re-analyzed with camera placement as a factor and it was confirmed that there was no substantive effect of such. Thus, fidgeting analyzes are reported using models associated with the fidgeting data for all participants.

## Results

The data were analyzed using linear mixed effects models (LMM) as implemented in *lme4* (Bates et al., 2012) in the R software environment. Given the ambiguity associated with estimating *p*-values for fixed effects in linear mixed models, we took as statistically significant those with |*t*| > 2 for all analyzes using a Gaussian distribution (see Baayen et al., 2008; Kliegl et al., 2010; Masson and Kliegl, 2013). For models using a binomial distribution (i.e., retention), *z* statistics and *p*-values are reported. Data are presented in Table 1. We chose to utilize linear mixed effect modeling for our analyzes, but the point should be made that alternative methods, such as structural equation modeling, or Bayesian graphical modeling, may be preferred by other researchers and suitable in future work.

**Table 1:** **Subject means and standard deviations (std.) as a function of block for attention ratings, fidgeting (micro and macro), and performance on the retention test**.

	**Block**							
	**1**	**2**	**3**	**4**	**5**	**6**	**7**	**8**
**ATTENTION RATING**								
Mean	6.0	6.3	5.9	5.9	5.4	5.2	5.2	4.9
Std.	1.0	1.0	1.0	0.7	1.1	1.1	1.2	1.0
**FIDGETING (MICRO)**								
Mean	12.2	10.0	12.1	11.9	11.4	11.1	12.1	14.0
Std.	8.0	7.0	8.1	6.7	6.9	6.0	8.5	9.1
**FIDGETING (MACRO)**								
Mean	4.8	4.2	4.6	6.2	5.7	4.3	5.8	7.5
Std.	3.6	3.6	3.9	4.9	3.9	3.0	4.2	4.5
**RETENTION TEST**								
Mean	58.3	77.7	71.2	64.8	49.1	49.0	82.3	55.3
Std.	29.6	38.5	33.9	32.6	46.7	37.4	25.2	39.9

### Influence of time on task (block)

Separate models were fit to the attention rating, fidgeting, and retention test data, all with block as a fixed effect and subject as a random effect. Furthermore, when analyzing fidgeting data, separate analyzes were conducted for micro and macro measures. The model fit to the retention test data used a logistic link function to capture the categorical (i.e., correct vs. incorrect) nature of the data while the attentional rating and fidgeting data were fit using the default Gaussian distribution.

The fixed effect of block was significant (i.e., |*t*| > 2) in the attention rating analysis. As block increased attention rating decreased, β = −0.18, *SE* = 0.03, *t* = −6.16. In the fidgeting analysis, micro fidgeting was not statistically related to block, β = 0.23, *SE* = 0.16, *t* = 1.49, however, macro fidgeting was, β = 0.30, *SE* = 0.10, *t* = 3.01, such that macro fidgeting increased as block progressed. The fixed effect of block was not significant in the analysis of retention test performance, β = −0.05, *SE* = 0.04, *z* = −1.16, *p* > 0.05. The latter analysis was conducted again with the data from block 7 removed. Retention test scores in block 7 were on average 30% higher than its neighboring blocks (i.e., block 6 and block 8). In addition, block 7 had the highest mean performance score observed across all blocks, but it was also associated with the lowest standard deviation, indicating uniformly high performance. Both of these descriptive statistics suggest the difficulty and/or nature of the retention test questions may have been different for this block relative to others. For example, two out of three questions drawn from block 7 contained material presented in the context of a humorous personal anecdote told by the instructor. Material can often be made more memorable when presented in the context of a personal story, particularly when humor is involved (Schmidt, 1994). We believe this may account for the increase in performance for this block relative to others in the second half of the experiment. In the model with block 7's data removed, the fixed effect of block was significant, β = −0.14, *SE* = 0.05, *z* = −2.93, *p* < 0.01, such that as block increased retention decreased. Furthermore, even while retaining block 7, and comparing the first vs. second half of the lecture (see Risko et al., 2012a, 2013a), retention was found to be significantly lower in the second half as compared to the first, β = −0.49, *SE* = 0.20, *z* = −2.39, *p* < 0.05. Note that in all subsequent retention test analyzes block 7 is omitted, however, parallel analyzes were conducted with this block included and where results diverge a note is made.

### Exploratory analysis: non-linear trends

The preceding analysis focused on the theoretically predicted linear effect of time on attention rating, fidgeting, and memory for lecture material. Inspection of the means across block, however, suggests the potential presence of non-linear trends in the data (see Table 1). To explore this possibility three additional models were fit to the attentional rating, fidgeting, and retention data with a set of four orthogonal polynomials as fixed effects (i.e., linear, quadratic, cubic, and quartic) and subject as a random effect. It is important to note that this analysis is exploratory, the intent of which is to provide potential fodder for future research into the influence of time in lectures.

In the analysis of attention rating the linear trend was significant such that attention decreased as block increased, β = −5.34, *SE* = 0.87, *t* = −6.17, but none of the higher order polynomial fixed effects were significant (all |*t*|'s < 2). In the analysis of macro fidgeting the linear trend was significant such that macro fidgeting increased as block increased, β = 8.86, *SE* = 2.88, *t* = 3.08, but there was also a significant quartic trend, β = 6.79, *SE* = 2.88, *t* = 2.36. There were no significant effects in the analysis of micro fidgeting (all |*t*|'s < 2). In the analysis of retention test performance the linear trend was significant such that retention decreased as block increased, β = −7.05, *SE* = 2.31, *z* = −3.06, *p* < 0.01, but there was also a significant cubic trend, β = 8.85, *SE* = 2.36, *z* = 3.76, *p* < 0.001. The addition of the retention data from block 7 altered this pattern such that only the quartic trend was significant, β = −11.42, *SE* = 2.48, *z* = −4.60, *p* < 0.001. This exploratory analysis suggests that, in terms of the relation between block and fidgeting and block and memory for lecture material, investigating non-linear trends might be theoretically fruitful.

### Relation between attention rating, fidgeting and memory for lecture material

In order to assess the relation between attention and fidgeting, two sets of LMMs were fit. In one set, retention test performance was the dependent variable with attention rating and fidgeting as predictors. In the second set attention rating was the dependent variable with fidgeting as the predictor.

In the analysis of the retention test performance, both macro fidgeting and attention rating were significant predictors. As attention rating increased so too did retention, β = 0.36, *SE* = 0.12, *z* = 3.00, *p* < 0.01, while increases in macro fidgeting were accompanied by decreases in retention, β = −0.10, *SE* = 0.04, *z* = −2.88, *p* < 0.01. Provided block was related to attention rating, macro fidgeting, and retention we re-ran the analysis with block included as a predictor. As in the previous analysis both attention rating, β = 0.30, *SE* = 0.13, *z* = 2.32, *p* < 0.05, and macro fidgeting, β = −0.09, *SE* = 0.04, *z* = −2.64, *p* < 0.01, were significant predictors. Interestingly, block was not significant in this model, β = −0.05, *SE* = 0.06, *z* = −0.90, *p* > 0.05, suggesting that the relation between block and memory for lecture material was mediated by one (or both) of the other predictors in the model (i.e., attention rating and/or fidgeting).

Using the micro instead of the macro fidgeting measure yielded the same pattern of results such that both attention rating, β = 0.41, *SE* = 0.12, *z* = 3.53, *p* < 0.001, and micro fidgeting, β = −0.05, *SE* = 0.02, *z* = −2.29, *p* < 0.05, were significant predictors of retention. When the retention data from block 7 was included rating remained significant, β = 0.36, *SE* = 0.11, *z* = 3.28, *p* < 0.01, but micro fidgeting did not, β = −0.03, *SE* = 0.02, *z* = −1.76, *p* > 0.05. Reverting back to the previous model, the addition of block did not alter the pattern of significance for attention rating, β = 0.34, *SE* = 0.13, *z* = 2.61, *p* < 0.01, nor micro fidgeting, β = −0.04, *SE* = 0.02, *z* = −2.11, *p* < 0.05. The addition of block was, as before, not a significant predictor, β = −0.06, *SE* = 0.05, *z* = −1.19, *p* > 0.05.

Exploring the relation between memory for lecture material and block further, a model with retention performance as the dependent variable and attention rating and block as predictors yielded a significant effect of attention but not block, β = 0.32, *SE* = 0.13, *z* = 2.42, *p* < 0.05, and, β = −0.08, *SE* = 0.05, *z* = −1.49, *p* > 0.05, respectively. In contrast, a model with retention performance as the dependent variable and macro fidgeting and block as predictors yielded significant effects, respectively, of both, β = −0.10, *SE* = 0.04, *z* = −2.73, *p* < 0.01, and, β = −0.11, *SE* = 0.05, *z* = −2.12, *p* < 0.05. In this case, the addition of block 7's retention data resulted in block no longer being significant, β = −0.02, *SE* = 0.05, *z* = −0.41, *p* > 0.05, while macro fidgeting remained so, β = −0.09, *SE* = 0.03, *z* = −2.83, *p* < 0.01. Reverting back to the previous model and replacing macro with micro fidgeting, block was found to be significant, β = −0.13, *SE* = 0.05, *z* = −2.70, *p* < 0.01, while micro fidgeting was not, β = −0.04, *SE* = 0.02, *z* = −1.87, *p* > 0.05. Neither factor was significant if the retention data from block 7 was included in the model, β = −0.04, *SE* = 0.04, *z* = −0.99, *p* > 0.05, and β = −0.03, *SE* = 0.02, *z* = −1.55, *p* > 0.05, respectively. This analysis suggests that the (linear) relation between block and memory for lecture material was mediated by attention.

In the analysis of attention rating, macro fidgeting was significant, β = −0.05, *SE* = 0.02, *t* = −2.18, such that as macro fidgeting increased attention rating decreased. Micro fidgeting did not share the same relation with attention rating, β = −0.001, *SE* = 0.01, *t* = −0.04. As above, given block was related to attention rating and macro fidgeting we re-ran the analysis with block included as a predictor. Interestingly, macro fidgeting was no longer significant, β = −0.02, *SE* = 0.02, *t* = −1.07, but block was, β = −0.17, *SE* = 0.03, *t* = −5.78. Thus, while macro fidgeting was related to attention rating this relation appears to be due to both being related to time on task (i.e., block).

## Discussion

The present investigation has provided a number of novel results with respect to attention, fidgeting, and memory for lecture material in a lecture-viewing task. Each of these results will be reviewed and the implications summarized below. In addition, we describe a non-attentional account of fidgeting that can explain the results reported.

### Time on task

The results of the present investigation demonstrated that fidgeting increased, while attention decreased, as a function of time on task. The latter result, that attention declines with time on task in a lecture environment, is consistent with previous research (Young et al., 2009; Risko et al., 2012a, 2013a) but extends it by analyzing a finer grain temporal distribution of attention probes (i.e., across eight blocks of 5-min intervals rather than dichotomizing lecture time into first and second halves) and employing a continuous measure of attentiveness (i.e., 7-point Likert rating rather than a dichotomous yes/no score on attention to the lecture). The former result, that fidgeting increases with time on task, is consistent with our prediction that as attention decreases with time into a lecture, fidgeting, as a potential route to enhance attention should increase, which is exactly what we found. This finding is convergent with previous studies that relied exclusively or partially on self-report measures of fidgeting (Galton, 1885; Mehrabian and Friedman, 1986; Gligoric et al., 2012; Carriere et al., 2013). Interestingly, this relationship with time was observed for macro but not micro fidgeting, providing tentative evidence for the existence of dissociable “types” of fidgeting. This possibility opens a potentially important line of inquiry for future investigation.

The results with respect to the influence of block on memory for lecture material were more equivocal. Overall there was no effect of block, however, this lack of an effect was found to be due entirely to the surprisingly accurate performance in block 7 (of 8), which appears to reflect the fact that the material covered in this 5-min section of the lecture was particularly memorable. Excluding the data from block 7 yielded an effect of time on task. An analysis comparing the first half of the lecture to the second also confirmed such an effect, even while retaining block 7's data. In both cases, participants remembered more from early in the lecture than later.

Also interesting with respect to time on task were the non-linear trends present in both the relations between fidgeting and time, and memory for lecture material and time. While these findings were derived from an exploratory analysis, it does suggest that a simple linear increase or decrease with fidgeting and retention for lecture material over time might be overly simplistic, and reinforces the use and analysis of our finer grained temporal slices in future research. Rather than speculate on the mechanism underlying these apparent non-linear trends, however, the first order of business for future research is to replicate the patterns reported here and then explore the mechanisms that subserve them.

As the data from block 7 makes very clear, while using naturalistic materials has its benefits in the study of attention (Kingstone et al., 2008; Risko et al., 2012b), when considering changes over time in actual lectures (whether linear or non-linear), it is important to note that the nature of the material presented also changes as a function of time (see Scerbo et al., 1992 for discussion of this issue). For example, there might be a general increase in the difficulty of the material discussed or random events in the lecture (e.g., a personal story) that might influence variables of interest (e.g., attention, fidgeting, memory for lecture material). Lecture material is often presented in a cumulative fashion in which subsequent material builds on that of previous with greater complexity (Scerbo et al., 1992), which may be of particular relevance for the general relationship between time and difficulty typically observed in a lecture context. These factors could lead to patterns of change over time that are specific to the lecture rather than some general underlying mechanism of, for example, attention function. Assessing the influence of time on task across many lectures (Young et al., 2009), or introducing controls in other ways (see Scerbo et al., 1992), is needed in order to derive general principles. That said, across a series of studies investigating mind wandering in lectures (Lindquist and McLean, 2011; Risko et al., 2012a, 2013a; Szpunar et al., 2013a,b), including the present investigation, there now appears to be some consistency with respect to attentional variations with time and the relation between mind wandering and memory for lecture material. The use of naturalistic lecture materials, of course, has the added benefit that the general patterns discovered should be applicable to actual lectures.

### Relation between attention, fidgeting, and memory for lecture material

In the present investigation we demonstrated that an individual's attention rating in a given block predicted their retention for lecture material during that block (i.e., as attention rating decreased retention decreased) consistent with prior research demonstrating a relation between attention and memory for lecture material (Lindquist and McLean, 2011; Risko et al., 2012a, 2013a; Szpunar et al., 2013a,b). This relation emphasizes the importance of research attempting to understand the factors which modulate attention in educational contexts (i.e., if one can engage a student's attention then one should be able to improve memory for educational material). A second interesting result with respect to the link between attention and memory for lecture material was that the linear effect of block on memory for lecture material (discussed above) was eliminated when an individual's attention rating was included in the analysis. This result suggests that the linear relation found between block and memory for lecture material was likely a function of block's relation to attention and the latter's relation to memory for lecture material. In other words, this analysis suggests that memory for lecture material declined as a function of time because attention was declining as a function of time, and attention modulates memory for lecture material. In addition to attention, fidgeting also predicted retention of lecture material as well, with retention declining as fidgeting increased. Interestingly, the relation between fidgeting and retention was significant even when attention rating was included in the model. This suggests that fidgeting makes a unique contribution to retention of lecture material over and above that contributed by an individual's attention. This result implies that fidgeting might be reflecting variation in some other encoding relevant mechanism. We speculate on this mechanism further below.

The idea that variations in fidgeting might be reflecting variation in some other encoding relevant mechanism draws additional support from the results of the analyzes relating attention to fidgeting. While the prediction that fidgeting would increase with time on task was confirmed, putatively supporting some relation between attention and fidgeting, when the contribution of time on task (i.e., block) to attention was controlled there was no relation between attention and fidgeting. This suggests that fidgeting does not explain any unique variance above and beyond what block explains in attention ratings. This fact will be important to consider in future research investigating the relation between fidgeting and attention (i.e., analyzes need to consider that both are related to time on task).

### Distribution of attention via self-reports

One interesting aspect of the attention rating response pattern related to its high mean value (5.6/7) relative to what might be expected based on prior research. Of 168 responses across our 21 subjects, only 9 of those responses indicated a value below a Likert response of 4 (which equated to varying strengths of disagreement with the statement that one's attention was fully focused on the video). While on its face this pattern might seem unusual given that prior mind wandering research typically finds rates of off-task responses between 30 and 40% (Smallwood et al., 2007b, 2008; McVay et al., 2009; Risko et al., 2012a, 2013a), it is important to note that participants were providing ratings that were meant to reflect a summary of their attentional state over the last 5 min. This needs to be contrasted with the point estimates provided by typical probe based analyzes of mind wandering. Thus, the overall “high” attentiveness is not necessarily inconsistent with prior estimates of the frequency of mind wandering if we suppose that over any given time period participants would be attending to the lecture 60–70% of the time. Alternatively, it remains possible that a regular reporting schedule encouraged more self-monitoring and improved attention to the lecture, which would have obvious practical benefits. If such continuous appraisal of one's attentional state (or some other aspect of the lecture; e.g., importance of material; Risko et al., 2013b) was demonstrated to reliably benefit the commitment of attention to the lecture material, then this could provide an inexpensive means to combat attentional lapses in a variety of contexts. It may be the case that such continuous monitoring could increase meta-awareness of both faltering attention or comprehension, as well as some of the more subtle cues (e.g., an increase in fidgeting) which may proactively indicate the onset of declining attention before a serious lapse actually occurs. While prior work has found no such benefit to self-monitoring in reducing mind wandering rates during a reading task in an experimental setting (Schooler et al., 2004), the additional intrinsic motivation present in an actual lecture context may interact with the efficacy of self-monitoring. Finding effective classroom interventions is frequently viewed as a priority for attention research, one such successful example being the application of mindfulness training. Such training has been shown to improve measures related to academic performance and appears to be mediated, in part, by reductions in mind wandering (Mrazek et al., 2013). Yet another promising form of intervention involves interpolating memory tests throughout the course of an online lecture, recently demonstrated to also be of benefit to educational outcomes (Szpunar et al., 2013a). See Szpunar et al. (2013b) for a review of interventions targeting mind wandering in educational contexts.

### Toward a non-attentional account of fidgeting

Taken together, the collective weight of the data suggests that while fidgeting changes as a function of time on task, and variations in fidgeting are related to memory for lecture material, these two patterns are independent of any strong relation between attention and fidgeting. This is inconsistent with the mechanism described in the introduction in which fidgeting might represent a means to increase attention (i.e., when individuals feel their attention waning they fidget in order to help them sustain attention). Nevertheless, an alternative view of fidgeting in the present context is that it reflects an individual's level of discomfort (see Galinsky et al., 1993). As noted in the introduction, listening to a lecture can be viewed as a sustained attention task. Critically, numerous studies have demonstrated that sustained attention (or vigilance) tasks are stressful (Temple et al., 2000; Grier et al., 2003; Warm et al., 2008a,b; Young et al., 2009). One line of evidence for increased stress during vigilance tasks comes in the form of elevated levels of catecholamines (Frankenhaeuser et al., 1971; Lundberg and Frankenhaeuser, 1980) and increases in self-reported distress (Szalma et al., 2004; Warm et al., 2008a). The primary source of this stress has been interpreted as relating to a common demand for the continual application of cognitive resources over a prolonged period (Warm et al., 2008a,b) and may therefore be generalizable to other contexts. The vigilance required during a lecture may thus be expected to induce stress for similar reasons. In the lecture context in particular, the increasing difficulty of the material as a lecture progresses (e.g., Scerbo et al., 1992) when combined with the vicissitudes of attention may lead to an increasing likelihood that the individual's situational model breaks down (Smallwood, 2011) which in turn could induce further stress as time on task increases. In addition to evidence suggesting that time on task is associated with stress in lectures, fidgeting is also known to be related to stress in that individuals tend to fidget more while under stress (e.g., Barash, 1974) and fidgeting appears to actually mediate the experience of perceived stress in some people (Mohiyeddini and Semple, 2013; Mohiyeddini et al., 2013). Lastly, while the relation between stress and memory is complex, induced stress can have a negative impact on memory (e.g., Kirschbaum et al., 1996; Payne et al., 2002). Taken together, these three observations provide a potential explanation for the patterns of fidgeting reported here in that (1) stress would be expected to increase with time on task, and thus (2) fidgeting should increase with time on task as observed, and (3) increases in fidgeting and thus stress should be negatively correlated with memory for lecture material. This stress account is speculative but it does dovetail with the current data. It also makes a directly testable prediction. Namely, if a “stress” rating was collected with each block rather than an attention rating, one should find that (a) self-reported stress should increase with time on task (b) variations in self-reported stress should be negatively correlated with variations in memory for the lecture material and finally (c) self-reported stress should be positively correlated with fidgeting. These predictions await empirical investigation.

## Conclusion

The present investigation has provided further evidence for the important role that attention plays in memory for lecture material. It has also highlighted the key role played by time on task as a modulator of students' attention in lectures. The present work has also provided an initial and objective consideration of fidgeting in the context of lecture viewing, and its relation to attention and memory for lecture material. The relationship we have shown between fidgeting and retention suggest that fidgeting may be an effective indicator of times when interventions (e.g., rest break or a change in lecture pacing) may be of particular benefit to educational outcomes. Additionally, the differential pattern of results across macro and micro levels of fidgeting implies that future research may benefit from examining correlates of different forms of fidgeting. This initial examination also strongly suggests that exploration of non-attentional views of fidgeting, in addition to attentional ones, will be a productive line of inquiry.

### Conflict of interest statement

The authors declare that the research was conducted in the absence of any commercial or financial relationships that could be construed as a potential conflict of interest.
